# Cord Blood Extracellular Vesicles Analyzed by Flow Cytometry with Thresholding Using 405 nm or 488 nm Laser Leads to Concurrent Results

**DOI:** 10.3390/diagnostics11081320

**Published:** 2021-07-22

**Authors:** Kristýna Pekárková, Jakub Soukup, Marie Kostelanská, Jan Širc, Zbyněk Straňák, Karel Holada

**Affiliations:** 1 Institute of Immunology and Microbiology, First Faculty of Medicine, Charles University, 128 00 Prague, Czech Republic; vrbova.k@gmail.com (K.P.); marie.kostelanska@lf1.cuni.cz (M.K.); Karel.Holada@lf1.cuni.cz (K.H.); 2 Department of Genetics and Microbiology, Faculty of Science, Charles University, 128 44 Prague, Czech Republic; 3 The Institute for the Care of Mother and Child, 147 00 Prague, Czech Republic; jan.sirc@upmd.eu (J.Š.); zbynek.stranak@upmd.eu (Z.S.)

**Keywords:** extracellular vesicles, flow cytometry, thresholding parameter, data correlation

## Abstract

Extracellular vesicles (EVs) from liquid biopsies are extensively analyzed by flow cytometry, a technology that is continuously evolving. Thresholding utilizing a violet 405 nm laser side scatter (VSSC) has recently been implemented. Here, we collected set of large EV (lEV) samples from cord blood, which we analyzed using a standard flow cytometer improved via a 405 nm laser side scatter. Samples were analyzed using two distinct thresholding methods—one based on VSSC, and one based on VSSC combined with fluorescence thresholding on stained phosphatidylserine. Through these thresholding methods, we compared lEVs from pre-term births and control cord blood. Double-labeled lEVs with platelet CD36+/CD41+, activated platelet CD41+/CD62P+ and endothelial CD31+/CD105+ antibodies were used. Apart from comparing the two groups together, we also correlated measured lEVs with the thresholding methods. We also correlated the results of this study with data analyzed in our previous study in which we used a conventional 488 nm laser SSC. We did not find any difference between the two cord blood groups. However, we found highly concurrent data via our correlation of the thresholding methods, with correlation coefficients ranging from 0.80 to 0.96 even though the numbers of detected lEVs differed between thresholding methods. In conclusion, our approaches to thresholding provided concurrent data and it seems that improving the cytometer with the use of a VSSC increases its sensitivity, despite not being particularly critical to the validity of flow cytometric studies that compare pathological and physiological conditions in liquid biopsies.

## 1. Introduction

There is an ongoing effort toward the standardization of flow cytometry analysis of extracellular vesicles (EVs), resulting in the publication of a joint position paper from the ISEV, ISAC and ISTH [1]. The MIFlow-Cyt EV establishes rules for EV measurement by flow cytometry, which allow for the reproduction of measured data in the future or by other groups using different cytometers. Additionally, efforts are being made to standardize the pre-analytical variables of EV flow cytometry measurement [2,3,4]. A collaborative work by 14 laboratories analyzed samples of well-defined platelet free plasma (PFP). However, significant variability remained [5,6]. This significant variability is caused by the varying sensitivities of cytometers. The conversion of arbitrary units to standard units helps to reduce this variability [7]. These combined efforts have resulted in the design of useful software tools, such as FCMPASS [8], which can assist with the reproducibility of EV measurements.

However, there remains a vast body of work that investigated EVs in blood by flow cytometry before any standardization efforts were made, such as the work surrounding MISEV in 2014 [9] and 2018 [10], as well as MIFlow-Cyt EV [1]. Here, we offer a unique comparison of thresholding on the same set of samples, employing one standard cytometer. We utilized secondary aliquots of frozen plasma samples from our current study, which used standard side scatter (SSC) thresholding on a 488 nm laser [11]. We upgraded our BD FACSCanto^TM^ II with a stronger 405 nm laser and equipped it with filters and mirrors for side scattering using this laser (VSSC), which improved the sensitivity of our cytometer. We also implemented thresholding with fluorescence (FITC labeled Lactadherin) in combination with VSSC. Fluorescence thresholding was reported to be more accurate and reproducible [12], but with a necessary “pre-selection” for phosphatidylserine (PS) [13]. On the other hand, contaminating events from buffers or lipoprotein particles were excluded [13].

Our data suggest that thresholding using a conventional 488 nm laser SSC or 405 nm VSSC leads to relatively similar results, although the absolute number of large EVs (lEVs) detected differs. Employing fluorescent thresholding with PS in combination with VSSC also leads to relatively similar results.

## 2. Materials and Methods

### 2.1. Samples

All patients signed informed consent agreements before sample collection. The study was approved by the ethical committee of The Institute for the Care of Mother and Child (no. 2015/06-02-4) and signed informed consent forms were obtained from all participants. A total of 19 samples of pre-term birth (gestation weeks 31.58 ± 2.52) cord blood and 10 samples of control (gestation weeks 38.10 ± 1.37) cord blood were collected for the study. All samples were collected from C-section births. Cord blood was collected in acid citrate dextrose solution an anticoagulant. Anticoagulated blood was centrifuged at 2800× *g* in a swing rotor at 24 °C for 15 min. Plasma was collected and centrifuged again under the same conditions. After the second centrifugation, aliquots of plasma were snap frozen in liquid nitrogen and stored at −80 °C.

### 2.2. Materials

Antibodies were purchased from Exbio: mouse anti human CD31 PerCP (IgG1, clone MEM-05); mouse anti human CD36 APC (IgG1, clone TR9); mouse anti human CD41 PE-Cy7 (IgG1, clone MEM-06); mouse anti human CD62 APC (IgG1, clone AK4); mouse anti human CD105 APC (IgG2a, clone MEM-226); mouse isotype control IgG1 APC (clone MOPC-21); mouse isotype control IgG1 PerCP (clone MOPC-21); mouse isotype control IgG1 PE-Cy7 (clone MOPC-21); mouse isotype control IgG2a APC (clone MOPC-173). Antibodies obtained from Santa Cruz Biotechnology: mouse anti human placental alkaline phosphatase (PLAP) PE (IgG2a, clone 8B6); mouse isotype control IgG2a PE. Bovine Lactadherin (Lact) FITC (BLAC-FITC) was acquired from Haematologic Technologies. ApogeeMix beads were obtained from Apogee Flow Systems (cat. no. 1493).

### 2.3. Sample Preparation

All samples were prepared in duplicate. Frozen plasma samples were thawed on ice and 40 µL aliquots were labeled with 10 µL of antibodies in the following combinations: CD36/CD41/Lact; CD41/CD62P/Lact; CD31/CD105/Lact; PLAP/CD105; IgG1 APC/IgG1 PE-Cy/Lact FITC; IgG1 PerCP/IgG2a APC/Lact FITC; IgG2a PE/IgG2a APC. Plasma with antibodies was incubated on ice for 30 min and then diluted with 1 mL of 0.1% BSA in PBS (PBS–BSA). Diluted samples were centrifuged at 20,000× *g*, at 4 °C for 20 min. The supernatant was removed and pelleted lEVS were diluted in 300 µL of PBS–BSA. Labeled samples were analyzed immediately.

### 2.4. Flow Cytometry Analysis

**Figure 1:**
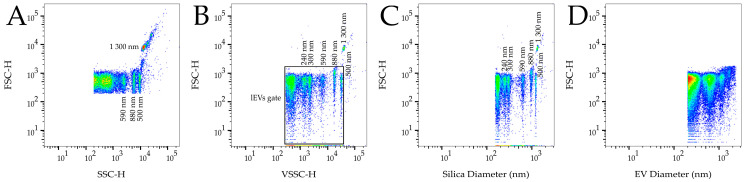
Representative density plots of ApogeeMix beads, which consist of 110 nm and 500 nm latex beads and 180 nm, 240 nm, 300 nm, 590 nm, 880 nm and 1300 nm silica beads. (**A**) Density plot measured on conventional 488 nm laser SSC-H with 4 distinguished populations of beads; (**B**) Density plot measured on 405 nm laser VSSC-H with 6 distinguished populations. Placement of lEV gate is shown; (**C**) Calibrated density plot with silica light scatter RI illustrating the calibration precision. The median value (in nm) of the 1300 nm beads is 1278, 880 nm—939, 590 nm—612, 300 nm—295, 240 nm—242. For 500 nm latex beads, the median value is 448 (for polystyrene RI); (**D**) Representative density plot of calibrated sample with EV diameter on X axis for high RI EVs. The size of the events in the lEV gate ranges from 200 to 2400 nm.

Samples were analyzed on a BD FACSCanto^TM^ II cytometer, improved by the use of a 100 mW 405 nm laser (run at 80 mW) and a 405/10 nm bandpass filter for the violet side scatter (VSSC). The VSSC threshold was set to 300 at 250 V, which is just below the signal of the smallest ApogeeMix beads (Figure 1B)—110 nm latex beads and 180 nm silica beads. The fluorescence threshold was set on the FITC channel (488 nm laser, filter 530/30) as 280 at 530 V. The fluorescence threshold was set (280 at 480 V) with VSSC (300 at 250 V) using the “AND” parameter (FITC + VSSC). The detector voltages were set as follows (installed filters in brackets): FSC—530 V; SSC—425 V (488/10); PE—350 (580/42); PerCP—650 V (670LP); PE-Cy7—675 V (780/60); APC—520 V (660/20). Each prepared duplicate was measured with each thresholding approach. Each sample was acquired 2 min at a low flow rate (measured value 9.2 µL/min). The ApogeeMix beads and the PBS–BSA buffer were measured on each measurement day.

### 2.5. Evaluation and Statistics

**Figure 2:**
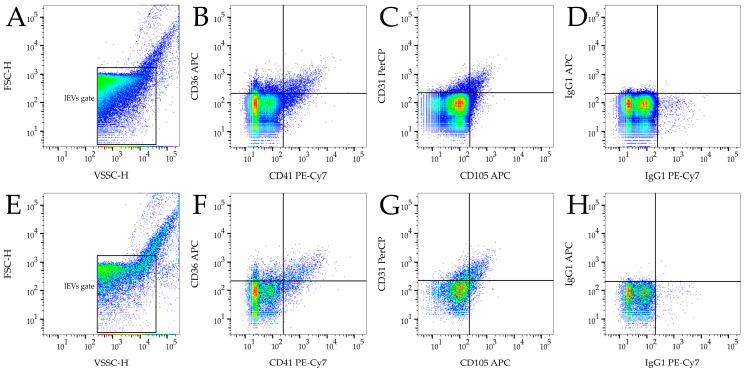
Gating strategy and representative density plots of flow cytometry lEV analysis. (**A**) Density plot of sample light scatter for VSSC threshold with lEV gate placing; (**B**,**F**) Illustrative density plots of CD36+/CD41+ lEVs for VSSC threshold; (**D**) Illustrative density plot of IgG1 PE-Cy7+/IgG1 APC+ for VSSC threshold. (**E**) Density plot of sample light scatter for FITC + VSSC threshold with lEV gate placing; (**C**,**G**) Illustrative density plots of CD31+/CD105+ lEVs for FITC + VSSC threshold; (**h**) Illustrative density plot of IgG1 PE-Cy7+/IgG1 APC+ for FITC + VSSC threshold.

FlowJo version 10.7.1 was used for sample analysis and gating. ApogeeMix beads were used for gate placement correction in each measurement (Figure 1B). The gate for lEVs was placed between the threshold level and the outer signal of 500 nm latex beads (Figure 1B). The same gate was used for samples measured with FITC + VSSC thresholding. Events in the lEV gate were analyzed using quadrant gating for fluorophores (Figure 2) and only double positive events were counted for further analysis. Fluorophore conjugates were chosen to avoid the need for fluorescence compensation.

GraphPad Prism 5 (ver. 5.03, GraphPad Software, San Diego, CA, USA) was used for the statistical analyses. The D′Agostino and Pearson omnibus normality test and the Shapiro–Wilk normality test were used to determine the normal distribution of the data. To compare the pre-term birth and control samples, two-tailed Mann–Whitney tests were used. For the purpose of the correlation of SSC, VSSC and FITC + VSSC thresholding methods, pre-term birth and control samples were compiled into one dataset. Linear regression analysis was used to determine the R2 of the linear trendline, followed by two-tailed Spearman correlation analysis (these data did not pass the normality tests). Data are presented as Spearman r values with 95% confidence intervals (CIs).

### 2.6. Calibration

FCMPASS software v. 3.07 [8] was used for the retrospective calibration of the SSC-H and VSSC-H data. The ApogeeMix beads size was calibrated using the manufacture provided refractive index (RI). To determine the diameter of the lEVs inside the acquisition gate, we utilized the software pre-defined high EV RI. Representative plots describing size of the beads and lEVs in nm are in Figure 1.

## 3. Results

Representative density plots of the analyzed ApogeeMix beads using SSC-H and VSSC-H are shown in Figure 1A,B, respectively. In the figure, distinct populations of 1300 nm, 880 nm, 590 nm silica beads and 500 nm latex beads are resolved on SSC-H, while 300 nm and 240 nm silica beads are also resolved on VSSC-H. The lEV gate placing used in the gating strategy is visible in Figure 1B. Figure 1C shows the ApogeeMix beads calibrated for the silica RI. The median values of the bead populations (in nm) correspond to their manufactured size. The representative calibrated density plot for lEVs is provided in Figure 1D. The diameters of the measured lEVs in the gate range from 200 to 2400 nm, approximately.

Illustrative light scatter density plots of isolated lEVs for VSSC and FITC + VSSC thresholding are shown in Figure 2A,E, respectively. The mean count of events detected in the lEV gate for VSSC was about eight times higher than in the FITC + VSSC thresholding method, both for pre-term and control samples (Table 1). As the control in each measurement, we analyzed the buffer used for dilutions. The mean background signal of the PBS–BSA buffer was 787 events/µL and 0.4 events/µL using VSSC and FITC + VSSC thresholding, respectively. The counts of lEVs for platelet CD36+/CD41+ were approximately 1100 and 800 for VSSC and FITC+VSSC, respectively (Table 1), activated platelet CD41+/CD62P+ counts were similar for both thresholding methods and endothelial CD31+/CD105 counts were about a quarter higher in the VSSC thresholding method (Table 1). No significant differences in the counts of platelet or endothelial lEVs between the pre-term and control groups were identified. Related isotype controls also did not show any significant differences (Figure 2D,H). We also included the detection of placental CD105+/PLAP+. Counts of lEVs were 260 ± 116 for pre-term samples and 452 ± 537 for control samples. However, the differences were not significant, and we omitted the results from further evaluation since we encountered a high variability in detected events in the IgG2a isotype control, suggesting nonspecific binding.

To confirm the presence of lEVs in our samples, we prepared samples for EM using lEVs isolated by an identical procedure used for flow cytometry analysis (Supplementary Figure S1). lEVs are visible in the overview image (Supplementary Figure S1a,b) and the lipid bilayer is visible in the detailed image (Supplementary Figure S1c,d).

**Figure 3:**
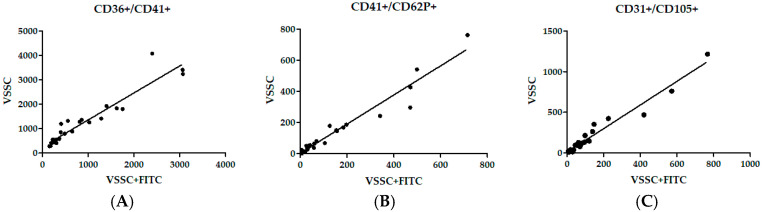
Representative correlation graphs of measured lEV counts with VSSC and fluorescence threshold (*x* axis) and VSSV-only threshold (y axis). (**a**) Correlation for CD36+/CD41+ double positive events; (**b**) correlation for CD36+/CD62P+ double positive events; (**c**) correlation for CD31+/CD105+ double positive events.

Each lEV sample duplicate was labeled and measured separately, and the results were used for the correlation analysis between the VSSC and FITC + VSSC thresholding methods. The correlation between the platelet counts of CD36+/CD41+, activated platelet CD41+/CD62P+ and endothelial CD31+/CD105+ lEVs is shown in Figure 3, with linear regression lines fitted on the graphs. Interestingly, a high level of correlation was achieved, even in comparison with data obtained in our previous study analyzing identical samples, which utilized conventional 488 nm SSC-H (Table 2). Goodness of fit of linear regression is represented as an R^2^ value ranging from 0.80 to 0.96, which means that there is a good fit for each comparison. Correlation statistics are represented by Spearman r values with 95% confidence intervals and *p* values. Each correlation was highly significant, with a *p* value < 0.0001. The correlation also ranged from 0.80 to 0.96, meaning that the measured data were related (Table 2).

## 4. Discussion

We utilized secondary aliquots from our previous study, comparing the presence of platelet and endothelial lEVs in the cord blood of pre-term and control newborns using conventional 488 nm laser SSC thresholding. We did not find significant differences in the counts of CD36+/CD41+, CD41+/CD62+ and CD31+/CD105+ lEVs [11]. Shortly after the analysis, we upgraded our cytometer with a stronger 405 nm laser and equipped it with mirrors and filters for VSSC. This allowed us to improve the sensitivity from about 400 nm to 200 nm lEV. For the calibration of the EVs’ diameter, we used the predefined settings of the FCMPASS software [8] for high-RI EVs since larger EVs (>200 nm) tend to have higher RIs [14]. The smallest resolved population of silica beads was 590 nm and 240 nm for 488 nm laser SSC and 405 nm laser VSSC, respectively. This enabled the incorporation of more EVs in the detection process [15]. We then analyzed frozen aliquots of collected cord blood plasma with the improved cytometer. Although this method meant that the cytometer had a better sensitivity and resolution, we again, did not see any significant differences in the numbers of detected lEVs between the studied groups. We also employed combined thresholding for VSSC and PS, detected by a lactadherin—FITC conjugate [16]. There was visible pre-selection by the FITC + VSSC thresholding method, which detected almost tenfold fewer total events, but also eliminated the detection of contaminating events from the buffer. Using VSSC, of the double positive platelet lEVs represented about 1.5% of all events in the lEVs gate, while with FITC + VSSC, the percentage increased to 8.5%. In absolute numbers, we detected about 70% of CD36+/CD41+ lEVs by FITC + VSSC compared to VSSC (1144 vs. 786) and 65% of CD31+/CD105+ lEVs (196 vs. 131). These results correspond with previously published data showing that most PS-positive EVs are from platelets [13], although their proportion does not correspond with the aforementioned data. We omitted CD105+/PLAP+ from the analysis due to the strong nonspecific binding of both antibodies of the IgG2a isotype in our experiments. The reason why IgG2a antibodies produce high nonspecific binding to lEVs is not known, but should be of interest in future studies. Double positive events were used to eliminate false positive events [17]. The counts of PS+ lEVs in pre-term newborns were modestly higher than in control samples (Table 1). No other differences in the counts of the studied lEV populations between the groups were found, which is in accordance with our previous study [11]. Interestingly, albeit the VSSC thresholding improved the size sensitivity and we utilized brighter fluorophores, the absolute numbers of the detected platelet or endothelial lEVs did not increase.

Since we analyzed samples using different thresholding methods, which led to similar results, we correlated both methods. Pre-term birth and control samples were combined together for the purpose of the correlation. We therefore found that both approaches correlated. In addition, we added the data from our previous study to the correlation, which were collected using conventional SSC [11]. Interestingly, these data also correlated to the VSSC and FITC + VSSC thresholding methods, despite the fact that different fluorophores were used on the markers. Most of the EVs in blood are smaller than 300 nm [18]. In our study, increasing the sensitivity of our cytometer or decreasing the number of detected EVs by fluorescence thresholding did not affect the overall results. Previous studies discussed the comparison of fluorescence with scatter thresholding [13] or VSSC with SSC [15], but the data were not correlated. Although the measuring of EVs is moving away from standard flow cytometry and toward nanoscale flow cytometry [19] or imaging flow cytometry [20,21], a great deal of studies have been carried out on conventional SSC or FSC thresholding. Our data suggest that these studies may obtain similar results if more sensitive VSSC or fluorescence thresholding methods are applied. It would be interesting to attempt the design of a similar study with a more sensitive cytometer that allows for the measurement of EVs up to 80 nm. Despite the differences in the counts of detected lEVs obtained via the methods we utilized in this study, we still acquired concurrent data, which we believe is an interesting outcome that provides valuable information for researchers.

## Figures and Tables

**Table 1:** Mean values of lEV counts and double positive lEV counts (lEVs/µL). * Significant differences *p* < 0.05.

Marker	Thresholding	Pre-Term	95% CI	Control	95% CI	*p* Value
lEVs	VSSC	78,932	74,618–83,245	75,607	69,769–81,446	0.4472
	FITC + VSSC	10,253	9355–11,511	8169	7491–8846	0.0438 *
CD36+/CD41+	VSSC	1155	663–1647	1133	468–1798	0.7655
	FITC + VSSC	855	457–1254	716	75–1357	0.5356
CD41+/CD62P+	VSSC	138	67–210	113	−53–279	0.1685
	FITC + VSSC	152	71–233	104	−53–261	0.0664
CD31+/CD105+	VSSC	169	81–256	223	−43–489	0.6629
	FITC + VSSC	102	41–164	159	−16–334	0.9451

**Table 2:** Correlation of measured double positive lEVs with different thresholding methods.

Marker	Thresholding Correlation	Linear Trendline (R^2^)	Spearman’s R	95% CI	*p* Value
CD36+/CD41+	SSC and VSSC	0.8086	0.8527	0.7015–0.9305	<0.0001
	SSC and FITC + VSSC	0.9195	0.8931	0.7784–0.9501	<0.0001
	VSSC and FITC + VSSC	0.9046	0.9488	0.8903–0.9765	<0.0001
CD41+/CD62P+	SSC and VSSC	0.8254	0.9322	0.8563–0.9687	<0.0001
	SSC and FITC + VSSC	0.9182	0.9352	0.8624–0.9701	<0.0001
	VSSC and FITC + VSSC	0.9457	0.9565	0.9064–0.9801	<0.0001
CD31+/CD105+	SSC and VSSC	0.8413	0.8336	0.6663–0.9210	<0.0001
	SSC and FITC + VSSC	0.7953	0.8034	0.6122–0.9059	<0.0001
	VSSC and FITC + VSSC	0.9593	0.9605	0.9148–0.9819	<0.0001

## Data Availability

The data used to support the findings of the presented study are available from the corresponding author upon request.

## Supplementary Materials

Figure S1 and related supplementary method is available online at https://www.mdpi.com/article/10.3390/diagnostics11081320/s1, Figure S1: EM pictures of lEVs centrifuged on grid.
