# Supplementary material for: Cord Blood Extracellular Vesicles Analyzed by Flow Cytometry with Thresholding Using 405 nm or 488 nm Laser Leads to Concurrent Results
Source: Diagnostics (Basel). 2021 Jul 22;11(8):1320. doi: 10.3390/diagnostics11081320 (PMC8392526; doi:10.3390/diagnostics11081320)
Supplement: Supplementary file 1 [file diagnostics-11-01320-s001.zip › diagnostics-1268702-supplementary.pdf]

Supplementary data:

Electron microscopy

Samples for electron microscopy (EM) were prepared to simulate the preparation of samples for flow cytometry. Briefly, plasma sample was diluted in PBS-BSA and centrifuged (Beckman Optima LE-80K, rotor SW40, 20 000 g, 4°C, 20 min) on formvar-carbon coated EM grids (Electron Microscopy Sciences, Hatfield, USA) [1]. The lEVs on the grid were fixed with 4% paraformaldehyde and 1% glutaraldehyde, contrasted with uranyl oxalate and embedded in methylcellulose as in [2].

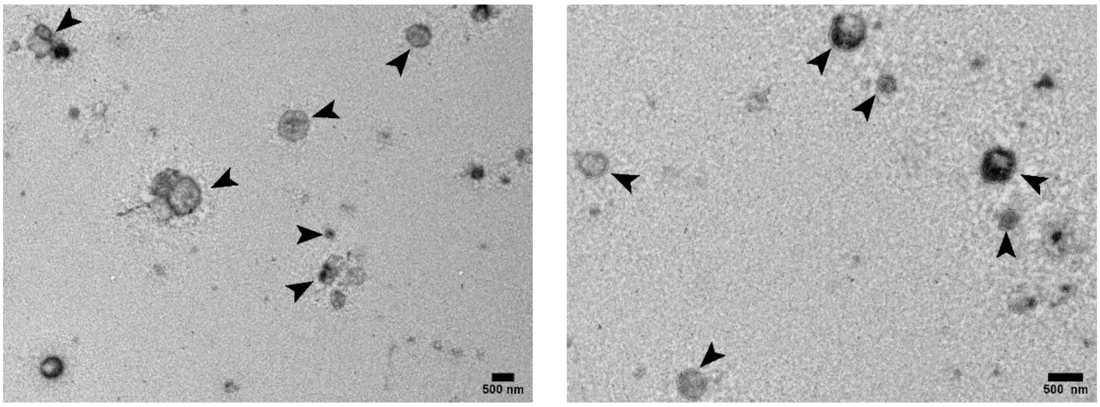

(**a**) (**b**)

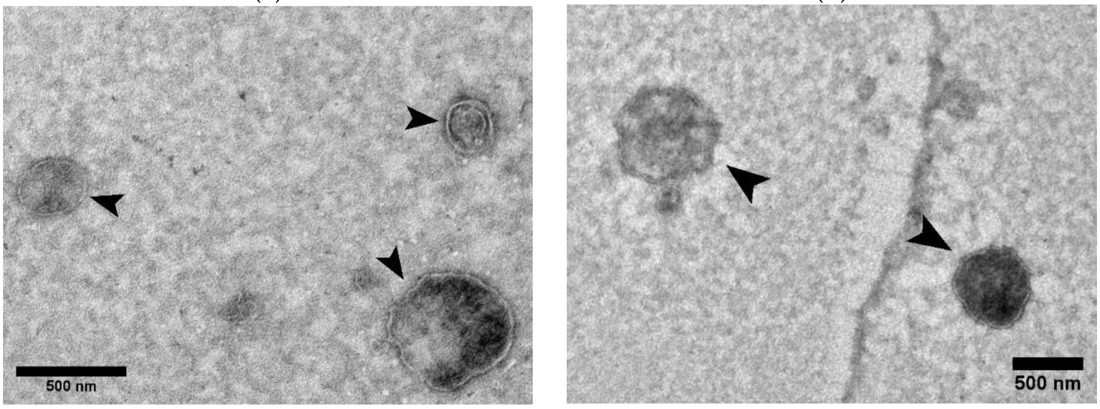

(c) (d)

Figure S1: EM pictures of lEVs centrifuged on grid (black arrowhead). (a and b) Overview pictures of lEVs sample and (c and d)) detailed picture of lEVs. Lipid bilayer vesicles of distinct sizes are visible in all pictures with minimum of other contaminating particles.

1. Arraud, N.; Linares, R.; Tan, S.; Gounou, C.; Pasquet, J.M.; Mornet, S.; Brisson, A.R. Extracellular vesicles from blood plasma: determination of their morphology, size, phenotype and concentration. *J. Thromb. Haemost.* **2014**, *12*, 614-627, doi:10.1111/jth.12554.
2. Théry, C.; Amigorena, S.; Raposo, G.; Clayton, A. Isolation and Characterization of Exosomes from Cell Culture Supernatants and Biological Fluids. *Current Protocols in Cell Biology* **2006**, *30*, 3.22.21-23.22.29, doi:https://doi.org/10.1002/0471143030.cb0322s30.
